# VEXAS syndrome: a new paradigm for adult-onset monogenic autoinflammatory diseases

**DOI:** 10.1007/s11739-023-03193-z

**Published:** 2023-01-20

**Authors:** Antonio Vitale, Valeria Caggiano, Antonio Bimonte, Federico Caroni, Gian Marco Tosi, Alessandra Fabbiani, Alessandra Renieri, Monica Bocchia, Bruno Frediani, Claudia Fabiani, Luca Cantarini

**Affiliations:** 1 Research Center of Systemic Autoinflammatory Diseases and Behçet’s Disease Clinic and Rheumatology-Ophthalmology Collaborative Uveitis Center, Department of Medical Sciences, Surgery and Neurosciences, University of Siena, Policlinico “Le Scotte”, Viale Bracci 1, 53100 Siena, Italy; 2 Hematology, Azienda Ospedaliera Universitaria Senese, University of Siena, Siena, Italy; 3 Ophthalmology Unit, Department of Medicine, Surgery and Neurosciences, University of Siena, Siena, Italy; 4 Medical Genetics, Department of Medical Biotechnologies, University of Siena, Siena, Italy; 5 Department of Medical Biotechnologies, Med Biotech Hub and Competence Center, University of Siena, Siena, Italy; 6 Genetica Medica, Azienda Ospedaliero-Universitaria Senese, Siena, Italy

**Keywords:** Monogenic autoinflammatory diseases, Diagnosis, Treatment, Haematology, Genetics, Ocular inflammation

## Abstract

VEXAS (Vacuoles, E1 enzyme, X-linked, Autoinflammatory, Somatic) syndrome is a recently described pathological entity. It is an acquired monogenic autoinflammatory disease caused by somatic mutations of the *UBA1* gene in blood cells precursors; the gene encodes one of the two E1 enzyme isoforms that initiates ubiquitylation in cell’s cytoplasm. VEXAS syndrome leads to systemic inflammation, with all organs and tissues potentially involved. The clinical picture may be extremely heterogenous, mimicking different other systemic rheumatologic entities coexisting with haematological disorders, especially myelodysplastic syndrome. This new disease represents a very intriguing clinical condition in several respects: it accounts for the paradigm of adult-onset monogenic autoinflammatory diseases determined by a genetic mosaicism resulting in the development of a challenging multiorgan inflammatory condition. Moreover, VEXAS syndrome is perhaps not an exceptionally rare condition and represents an example of a systemic genetic autoinflammatory disease drawing its origin in bone marrow disorders. VEXAS syndrome should be strongly considered in each adult patient with an unexplained systemic inflammatory condition, especially when recurrent fevers, neutrophilic dermatosis, relapsing polychondritis, ocular inflammation and other systemic inflammatory symptoms accompanying myelodysplastic syndrome or other haematological disorders. The syndrome deserves a multidisciplinary approach to reach the diagnosis and ensure the best management of a potentially very challenging condition. To quickly describe the clinical course, long-term outcomes, and the optimal management of this new syndrome it is essential to join forces internationally. To this end, the international AutoInflammatory Disease Alliance (AIDA) registry dedicated to VEXAS syndrome has been developed and is already active.

## Introduction

VEXAS (Vacuoles, E1 enzyme, X-linked, Autoinflammatory, Somatic) syndrome is a new pathological entity first reported in December 2020. VEXAS is an acquired autoinflammatory disease caused by somatic mutations of the *UBA1* gene; it is characterized by a severe adult-onset chronic inflammation generally associated to haematological disorders. It opens new wide scenarios about the understanding of acquired monogenic adult-onset autoinflammatory diseases and the identification of adult-onset inflammatory disorders related to innate immunity [1].

The interest about this new clinical entity has been evident as soon as the first report in scientific literature was published, with a wide number of publications reported as soon as the first 21-month period (124 papers on PubMed). This is mainly due to the interest on how disorders of bone marrow progenitors may promote severe systemic inflammation, and to the peculiarity of *UBA1* mutations in inducing an adult-onset autoinflammatory disease through a genetic mosaicism. Furthermore, the unexpected number of VEXAS patients identified in a short time seems to suggest a considerable epidemiological relevance in the coming years.

In this context, the purpose of this review is to summarise the scientific evidence accrued from the first description of VEXAS syndrome, highlighting what is currently known about genetic, clinical, laboratory, diagnostic and treatment features of this new autoinflammatory disease.

## Methods

An extensive literature search in the Medline database (via PubMed) was performed up to 30 September 2022. We searched for published evidence using “VEXAS syndrome”, “VEXAS” and “VEXAS disease” as keywords for research. Papers published in English from December 2020 were screened for eligibility based on title, abstract, and keywords. Information from studies based on wide numbers were all included in this review, while case series and case reports were included when they added practical information or induced to speculate about specific aspects of VEXAS syndrome. Case reports were also considered when they described remarkable information not reported elsewhere, to provide a wider comprehension of the disease. As a whole, 8 case series and 16 case reports were included in the review because they were considered to add additional information; 8 case series and 36 case reports were ruled out as considered to add a few more evidence.

## Genetic features

VEXAS syndrome is caused by somatic postzygotic mutations of the *UBA1* gene located on chromosome Xp11.23. Up to December 2020, the *UBA1* (Ubiquitin-Like Modifier-Activating Enzyme 1), encoding one of the two E1 enzyme isoforms that initiates ubiquitylation in cell’s cytoplasm, had been associated to the X-linked infantile spinal muscular atrophy in patients with germline mutations. Conversely, acquired *UBA1* gene mutations in blood cells precursors are associated to a specific and unique systemic inflammatory entity [1]. This syndrome is mostly observed in males because of the X-linked expression; nevertheless, X0 monosomy may explain some female cases, as for a 67 years-old patient with Turner syndrome described by Stubbins et al. [2]. Of note, also female VEXAS patients without X chromosome monosomy or apparent microdeletions have been identified [3]. In this regard, whether the skewed X-chromosome inactivation may enhance the role of *UBA1* gene mutations on the second X chromosome is matter of debate, as previous studies have shown that *UBA1* figures among the genes not subjected to X-inactivation [4].

The most frequently reported mutations of the *UBA1* gene affect the methionine at amino acid position 41. At current, p.Met41Thr(c.122T > C), p.Met41Val(c.121A > G), and p.Met41Leu (c.121A > C) account for the most frequently pathogenic variants; the p.Ser56Phe (c.167 C > T) and p.Gly40_Lys43del (c.118–1G > C), respectively likely pathogenic and pathogenic, have also been identified in patients with no p.Met41 mutations, but presenting a typical VEXAS phenotype [5, 6]. The mutation Gly40Ala has been observed associated to the p. Met41Leu in some cases [7, 8]. Among other mutations, p.Met41Val variant seems to represent a risk factor for decreased survival in VEXAS syndrome. Indeed, the p.Met41Val variant has been found to cause a lower translation of the catalytically proficient UBA1b isoform when compared to the p.Met41Leu or p.Met41Thr mutations [7].

To characterize the pathogenic role of mosaicism Beck et al. [1] isolated and sequenced different hematopoietic cell populations. They discovered that patients with somatic *UBA1* mutations presented predominantly wild-type T and B lymphocytes and predominantly mutant myeloid cells (neutrophils and monocytes) in the peripheral blood. Oppositely, both lymphoid and myeloid progenitors (multipotent progenitors, granulocytes-monocytes progenitors, megakaryocyte-erythroid progenitors) had abundant mutant cells in the bone marrow. A lower proliferation or a depletion of the mutant lymphoid cells could explain the absence of mature mutated lymphocytes in the peripheral blood.

Of note, other somatic mutations may coexist with *UBA1* variants, especially in *DNMT3A* (9.2–22% of cases) and *TET2* (5–11% of cases) genes [5]. The main mutation on *DNMT3A* is p.Arg882His (c.2645G > A) [6]. In particular, a loss-of-function of *DNMT3A*, which acts as a repressor of inflammation, may further contribute to the proinflammatory status of VEXAS syndrome. In this regard, the deficiency of this protein has been related to the activation of innate immune inflammatory signalling in myeloid cells. Mutations in *DNMT3A* seem to provide a competitive advantage to *UBA1* mutated cells undergoing an inflammatory overstress, due to a sort of resistance to apoptosis in hematopoietic progenitors [9]. More recently, an *ASXL1* coexisting mutation has been detected [3]. These coexisting mutations could represent coincidental age-related findings whose significance deserves in-depth studies.

According with Obiorah et al., half of the 6 patients complicated with myelodysplastic syndrome (MDS) had abnormal cytogenetics including del5q and del13q in a first patient, del20q in a second patient and t(3;12) in a third patient. At current, *GNA11*, *CSF1R*, *EZH2* represent other genes associated to hematologic malignancies in VEXAS patients complicated with MDS [10].

## Immunologic features

At transcriptome analysis of the peripheral blood, a shared gene expression signature consistent with the activation of multiple innate immune pathways has been highlighted in VEXAS patients. In details, monocytes and neutrophils showed a highly activated inflammatory signatures in multiple pathways with hyper-expression of tumour necrosis factor (TNF), interleukin(IL)-6, IL-8, and interferon(INF)-γ, which is consistent with cell-intrinsic severe myeloid inflammation. This was also confirmed by the assessment of gene expression patterns and by the increasing of serum cytokines levels in peripheral blood. In line with these results, CRISPR–Cas9-edited zebrafish knockout of UBA11b, a homologue isoform of the human UBA1, showed a gene upregulation of pro-inflammatory cytokines including TNF, IL-6 and IL-8.

Mutant neutrophils have a preserved phagocytic capacity, but an enhanced spontaneous neutrophil extracellular trap formation, which proves a proinflammatory neutrophil activation in VEXAS patients. Pathways affecting the unfolded protein response and an integrated stress response were also identified although limited at myeloid cells [1, 6].

The p.Met41 mutations have been found to induce a loss of cytoplasmic UBA1 function by generating a catalytically deficient isoform termed UBA1c. Looking at mutated monocytes, decreased levels of the catalytically proficient UBA1b isoform and detectable levels of UBA1c were observed, with consequent reduction of ubiquitylation activity. By contrast, peripheral T cells (which carries the *UBA1* mutation in a small percentage) showed no differences in proficient UBA1 isoforms when compared to T cells from unaffected individuals [1, 11]. Jachiet et al. reported a significant decrease in peripheral dendritic cells and in monocyte subsets in blood from VEXAS patients with MDS, perhaps due to a redistribution of these cells into inflammation sites, or to an increased apoptosis, or an impaired bone marrow production [12].

Interestingly, human leukocyte antigens (HLA) polymorphisms, especially the B27 and B51 haplotypes, have been proposed to influence the clinical presentation of VEXAS patients [13]. This hypothesis is merely based on a case report and should be deepened with basic research and a larger number of patients.

## Clinical features

**Fig. 1:**
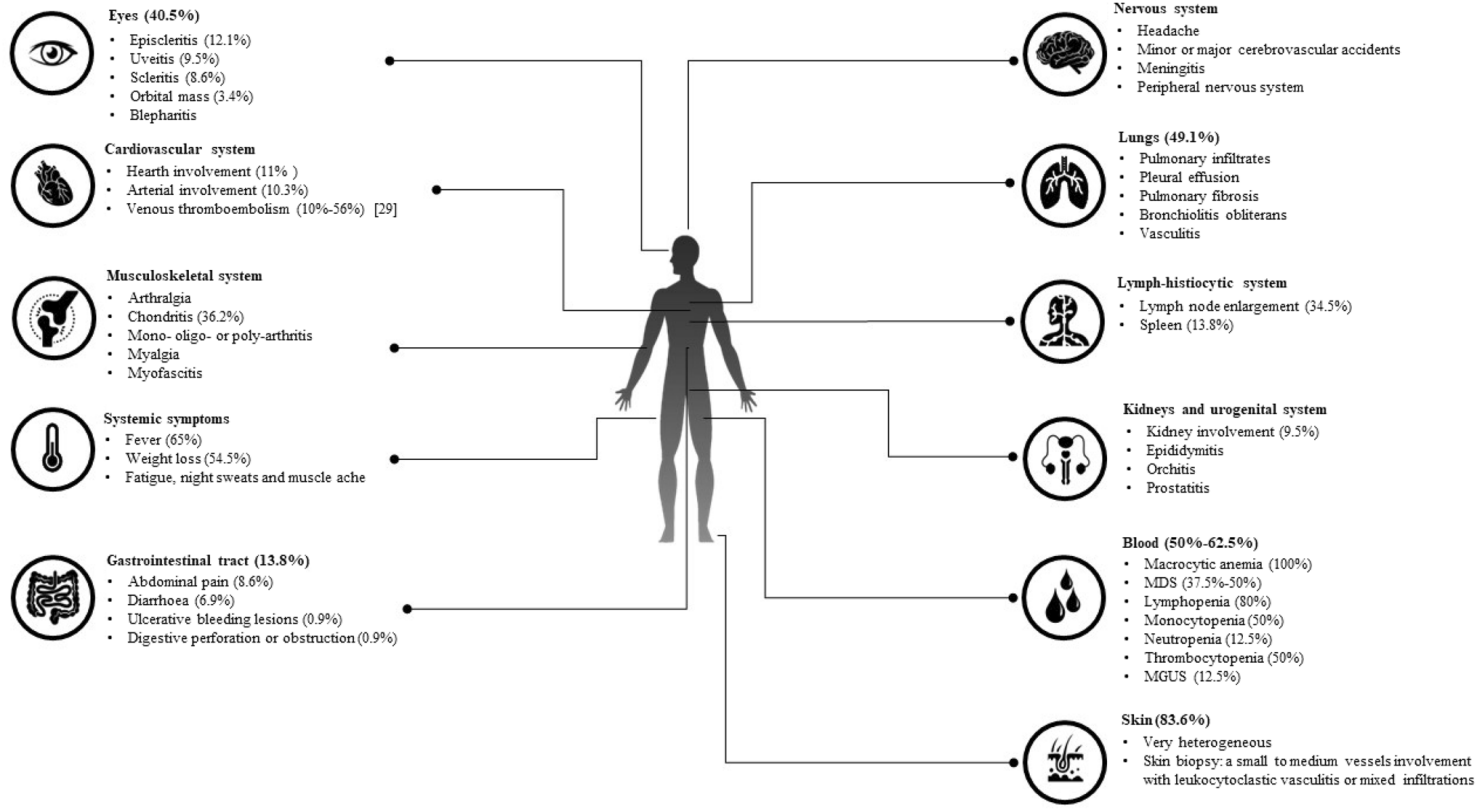
The cartoon describes the main clinical and laboratory hematological manifestations in VEXAS patients. Frequencies are driven from literature, especially Georgin-Lavialle et al. [5], who described the widest number of VEXAS patients so far, Obiorah et al. [10], who described the haematological involvement in 16 patients with VEXAS syndrome, and Groarke et al. [29], who deeply described thromboembolism in VEXAS syndrome

Since all organs and tissues may be involved by inflammation, the clinical picture of VEXAS syndrome is extremely heterogeneous. Actually, VEXAS manifestations may mimic different systemic rheumatologic disorders associated to MDS, including small vessels vasculitides, rheumatoid arthritis, seronegative spondyloarthritis, Sweet syndrome, relapsing polychondritis (RP), polyarteritis nodosa, and even Behcet’s disease [1, 14]. Figure 1 describes the main clinical manifestations observed in VEXAS syndrome with the corresponding frequency driven from literature [5].

According to Georgin-Lavialle et al., VEXAS syndrome occurs mostly in male adults from 40 years onwards, with a median age of 67 years at the time of the symptomatology onset. It shows up with constitutional symptoms, such as fever and fatigue, and other clinical and laboratory clues including neutrophilic dermatosis, chondritis, vasculitis, pulmonary infiltrates, cytopenia, dysplastic bone marrow. The disease often shows a treatment-refractory course associated with a high mortality rate.

### Systemic symptoms

They are very common and consist of fever (roughly 65% of patients), weight loss (55% of patients), fatigue, night sweats, muscle ache [5].

### Blood

A haematological involvement manifests with a MDS in more than a half of the patients. The MDS subtypes include the form with ring sideroblasts or with multilineage dysplasia. When comparing patients with VEXAS-MDS syndrome and subjects with VEXAS syndrome alone, the former shows most commonly non-infectious recurrent fevers, gastrointestinal tract involvement, pulmonary infiltrates, and arthralgia. These patients have lower platelet count and require glucocorticoid treatment more frequently. A small percentage of the patients with MDS also develops a monoclonal gammopathy with unknown significance (MGUS) [5, 10].

According with Obiorah et al., macrocytic anemia was observed in all 16 patients enrolled despite the normal vitamin B12, folate and copper levels, while thrombocytopenia was identified in 8 out of 16 patients. Absolute lymphopenia and monocytopenia were noted in 80% and 50% of patients, respectively, while neutropenia was less common, with 2/16 (13%) of patients involved. Circulating immature granulocytic precursors were found in 11 of 16 patients [10]. On review of peripheral smears, 10 patients had at least one among cytoplasmic vacuoles, hypogranular or hyposegmented neutrophils and pseudo Pelger–Huët-like anomaly. In particular, vacuolated monocytes were observed in 9 patients [10].

In the case series described by Obiorah et al., MDS was diagnosed in 6/16 (38%) patients, 4 of which suffered from MDS with multilineage dysplasia and 2 from MDS with single lineage dysplasia. Median time from symptom onset to MDS was 5.0 years; 5/6 (83%) patients were dependent on red blood cells (RBC) transfusions, and 1 patient showed a worsening anemia despite erythropoietin-stimulating agents. In contrast, only 4 (40%) of 10 of those who did not meet WHO criteria for MDS were dependent on RBC transfusions. Two of the 10 patients without MDS were also diagnosed with multiple myeloma [10]; other two patients were diagnosed with chronic lymphocytic leukemia at flow cytometry.

Hage-Sleiman et al. reported a 67-year-old man with a 10-year history of essential thrombocythemia associated to a *CALR* mutation and skin infiltration of non-blastic tumor cells (otherwise defined “myelodysplasia cutis”) associated to ad *UBA1* mutation. Both the CALR clone and the essential thrombocythemia phenotype disappeared at the time of the identification of the *UBA1* gene mutation. This induced the authors to suggest that *UBA1* mutations could provide a powerful selective advantage capable to overcome CALR mutated clones. However, this observation could be coincidental or induced by the use of hydroxyurea and thalidomide; therefore, further observations should be obtained to corroborate the authors’ hypothesis [15].

Interestingly, the frequency of VEXAS syndrome in male patients with a confirmed myeloid malignancy and systemic inflammatory and autoimmune diseases has been reported to be of 12% [16]; the frequency of VEXAS syndrome among male and female patients with unexplained peripheral cytopenia was identified at 0.66% [3].

Cases of VEXAS patients complicated with macrophage activation syndrome and hemophagocytic lymphohistiocytosis have also been reported. In particular, macrophage activation syndrome could be a direct consequence of the defective ubiquitylation causing the inflammation. Moreover, the altered ubiquitin homeostasis can be enhanced by macrophages able to activate innate immune pathways [17, 18].

### Skin

**Fig. 2:**
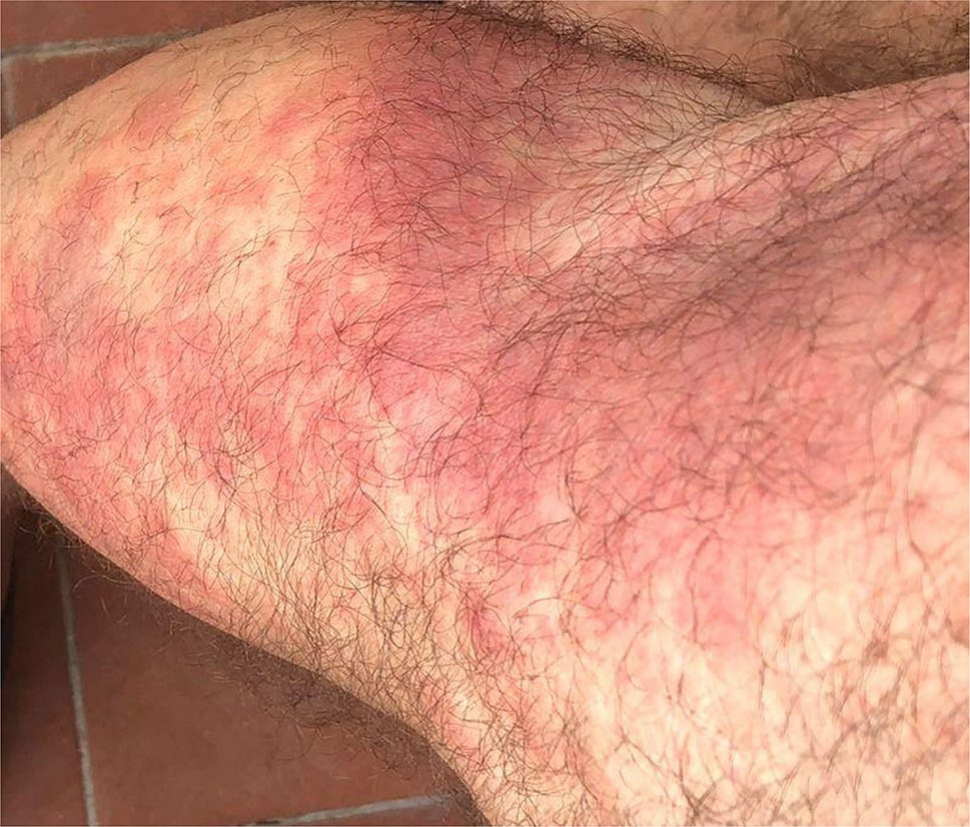
Erythematous plaques in the right lower limb in a patient with VEXAS syndrome

The skin involvement is the most frequently encountered (up to 84% of the patients) and can be very heterogeneous. It can show up with a neutrophilic dermatitis, vasculitic features, erythematosus papules, erythema nodosum, urticaria, periorbital oedema (seldom, described as nummular and violaceous), injection-site reactions (especially after anakinra administrations), in descending order of frequency [5, 19]. In addition, multiple, firm, tender, infiltrated erythematous or violaceous Sweet syndrome-like nodules on the trunk, limbs, and neck have been described [19]. Other manifestations also reported are oedematous, erythematosus, violaceous, purpuric, or urticarial plaques, purpuric macules, eczematous rash, bullae, subcutaneous nodules, morbilliform maculopapular exanthema, erythema exudativum multiforme [1, 17, 20–26]. Figure 2 shows erythematous plaques affecting the lower limbs in a patient with VEXAS syndrome.

A skin biopsy can show a small to medium vessels leukocytoclastic vasculitis or mixed infiltrations with lymphocytes, neutrophils, and eosinophils; in other cases, a single-cell type infiltrate may be observed. Less frequently, monocytic or histiocytoid dermal infiltrates can be observed in VEXAS patients and lymphocytic vasculitis may be described on cutaneous pathology. Skin nodules with lymphocytic infiltrate and no evidence of neutrophils, eosinophils or vasculitis has been also reported in a VEXAS patient [27].

Panniculitis or cholesterol emboli of medium vessel artery are further possible histologic manifestations [1, 17, 20–26]. Many cells in the infiltrate appeared to be CD68-positive, myeloperoxidase-positive ‘‘histiocytoid’’ myeloid precursor cells similar to that detected in Sweet syndrome [19]. Through paired sequencing analysis, it has been demonstrated that the dermal infiltrates in VEXAS syndrome derive from the same pathological *UBA1*-mutated myeloid clone found in the bone marrow [23].

### Eyes

**Fig. 3:**
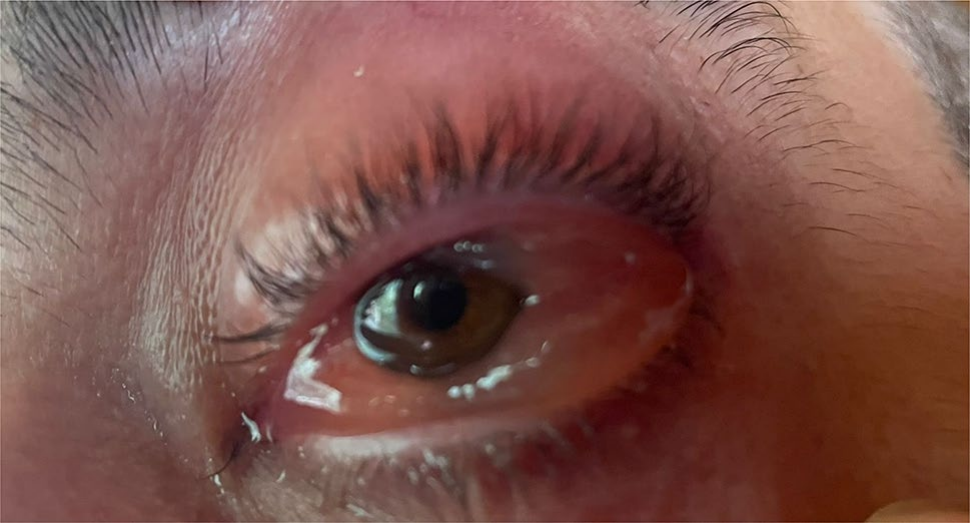
Orbital inflammation associated to conjunctival chemosis and eyelid oedema are observed in the left eye from a VEXAS patient

Ocular involvement occurs in up to 40.5% of the cases, mostly with episcleritis (12.1%), uveitis (9.5%), scleritis (8.6%), orbital mass (3.4%) and blepharitis. Orbital and periorbital inflammation may occur [28]. Figure 3 shows eye involvement in a VEXAS patient suffering from orbital inflammation associated to conjunctival chemosis and eyelid oedema.

### Cardiovascular system

Heart involvement (in about 11% of patients) can show up with pericarditis, myocarditis, and possibly evolve in a cardiomyopathy [5].

Arterial involvement (10.3%) with aortitis (1.7%), aneurysms (3.4%) and anti-neutrophil cytoplasmic antibodies (ANCA)-negative vasculitis have to be ruled out. Episodes of venous and arterial thrombosis in various districts have been reported in roughly one third of the patients, with the 60% of them occurring during the first 2 years from the symptomatology onset [5]. The prevalence of venous thromboembolism (VTE) is reported between 10 and 56% of cases [29]. Obiorah et al. found out 9 VEXAS patients with VTE, 2 of which had high factor VIII levels, which is associated to increased risk of venous thrombosis via enhanced thrombin formation; 1 patient had a high factor IX serum level [10]. Unlike VTE, arterial thrombosis appears to be less common (< 10% of cases reported) [29]. Based on the frequency of venous thrombosis, some authors have also suggested to think about VEXAS syndrome in all men with unprovoked VTE associated to systemic inflammatory manifestations and macrocytic anemia or thrombocytopenia [30].

Two studies evaluated the presence of antiphospholipid antibodies. Beck et al. reported the presence of lupus anticoagulant in 12/25 (48%) patients with VEXAS syndrome [1]. Obiorah et al. reports that 7/16 (44%) patients had persistently positive lupus anticoagulant and 5 (55.5%) of 9 patients with VTE had positive LA. One patient also had weakly positive IgM anticardiolipin antibodies [10].

Analysis of the transcriptome on VEXAS neutrophils demonstrates an upregulation of gene networks in proinflammatory cytokines, tumour necrosis factor, interleukin-6, interferon-γ, complement, coagulation, hypoxia, JAK/STAT, and reactive oxygen species amongst others relevant to thromboinflammation [1, 29].

Primary neutrophils from patients with VEXAS demonstrated exaggerated spontaneous formation of the neutrophil extracellular traps (NET), in the absence of an exogenous stimulant. NET is a particular inflammatory form of cell death characterized by extracellular release of DNA as a web-like scaffold. NETs support thrombogenesis mediating a scaffold function for platelet adhesion, coagulation factor presentation, and fibrin formation [29].

All these reasons leading to VTE should induce to consider an anti-thrombotic prophylaxis in all VEXAS patients, especially those with additional high-risk pro-thrombotic factors such as immobility or recent surgery. Future studies will highlight the more suitable prophylaxis [29], which must consider also an increased risk of bleeding in such patients, at least partially explained with thrombocytopenia and the use of non-steroidal anti-inflammatory drugs (NSAIDs) for disease control.

### Lungs

Lung involvement is very common (about half of the patients) with pulmonary infiltrates and pleural effusion accounting for the most frequent disease manifestations. Pulmonary fibrosis, bronchiolitis obliterans and vasculitis have also been described in VEXAS patients [1, 5, 24].

### Gastrointestinal tract

Gastrointestinal involvement is not common and affects only 13.8% of the patients. The most common symptom is abdominal pain (8.6%), followed by diarrhoea (6.9%), ulcerative lesions with gastrointestinal bleeding (0.9%) up to digestive perforation or obstruction (0.9%) [5]. Perforation of the jejunum during treatment with the anti-IL-6 tocilizumab has been described [22].

### Kidneys and urogenital system

Kidney involvement has been observed in 9.5% of the patients [5]. It occurs with proteinuria, erythrocyturia with dysmorphic erythrocytes and progressive renal disease up to kidney insufficiency. A renal biopsy revealing endotheliitis with medium-sized vessel vasculitis and interstitial infiltrate of myeloperoxidase and CD68 positive myeloid cells has been reported [22].

Epididymitis is common in VEXAS patients, along with orchitis or prostatitis.

### Nervous system

Headache, minor or major cerebrovascular accidents, meningitis, and peripheral nervous system involvement with sensory neuropathy (5.2%) and multiple mononeuropathy (2.6%) are observed [5]. Of note, a case of chronic inflammatory demyelinating polyradiculoneuropathy has also been reported; nerve biopsy showed a demyelination process with thin myelin sheaths and concentric proliferation of Schwann cells in addition to axonal loss [31].

### Musculoskeletal system

Arthralgia, mono-, oligo- or poly-arthritis and myalgia are often reported. Intriguingly, up to 50% of the patients may develop chondritis of the ears, nose, and other cartilages [5].

According with the retrospective study by Ferrada et al., 7 out of 92 (7.6%) subjects diagnosed with RP based on internationally accepted criteria showed a p.Met41 mutation on the *UBA1* gene. Notably, none of the patients with VEXAS-associated RP (VEXAS-RP) presented chondritis of the airways or costochondritis. Mortality was higher in VEXAS-RP patients than in patients with no *UBA1* mutations (27% vs 2%). Similarly, elevated acute phase reactants and hematologic abnormalities were prevalent in VEXAS-RP subjects. Based on these findings, an algorithm aimed at distinguishing patient with VEXAS-RP from patients with RP without VEXAS was provided. The algorithm tree is composed by male sex at the first node, by mean corpuscular volume > 100 fL at the second node and platelet count < 200 k/µL at the third node. All patients fulfilling the three nodes were correctly identified with VEXAS-RP and three patients with RP were incorrectly classified as VEXAS-RP. Therefore, the algorithm showed a 100% sensitivity and a 96% specificity [32]. Later, a retrospective study comparing 95 VEXAS-RP patients with 40 patients suffering from idiopathic RP confirmed that the male sex, older age, and the presence of fever, skin manifestations, ocular involvement, pulmonary infiltration, heart inflammatory affections, were more frequent in VEXAS patients [33].

Extensive and severe myofascitis characterised by perimysial, epimysial and fascial macrophagic CD68 positive inflammation with moderate necrotizing myopathy at histology has been described in VEXAS patients. Noteworthy, prominent intramuscular non-red-rimmed vacuoles were noted [8].

### Lymph nodes

Lymphadenopathy is common (about 35% of patients), especially in hilar and mediastinal lymph nodes, but also in cervical, axillary, abdominal and inguinal sites. Spleen is described enlarged in 13.8% of the patients [5].

## Diagnosis

Diagnosis of VEXAS syndrome is based on the right interpretation and combination of clinical manifestations and laboratory features, followed by a multidisciplinary approach including, among others, rheumatologists, immunologists, haematologists, geneticists, pathologists, ophthalmologists, dermatologists, internal medicine specialists, geriatricians, and radiologists. A definite diagnosis is obtained with the identification of *UBA1* gene mutations; however, the observation of vacuoles in bone aspirate smear along with the identification of MDS or other haematological disorders at laboratory assessment or bone marrow biopsy represent essential steps toward diagnosis.

### Laboratory features

Serological investigation generally shows elevated inflammatory markers levels (as for erythrocyte sedimentation rate and C-reactive protein), macrocytic anemia (with normal copper, B12 and folate levels) combined with an inflammatory anemia. In particular, VEXAS syndrome is frequently associated to unexplained macrocytosis, but anemia with normal mean corpuscular volume may be also observed in some cases [3]. Electrophoresis of plasma proteins eventually followed by serum immunofixation allow the identification of MGUS or multiple myeloma, which may accompany VEXAS syndrome [10]. Ferritin serum levels may be moderately increased, without reaching the markedly increased values typically observed in patients with Still’s disease [22].

Thrombocytopenia and leukopenia may be also found, especially as lymphopenia and monocytopenia. No disease-specific autoantibodies are usually found, but lupus anticoagulant and anti-cardiolipin autoantibodies have been observed [1, 10].

According to Obiorah et al., CD19 B cells resulted to be decreased in 8/12 (66.7%) patients in peripheral blood, whereas natural killer cells were found decreased in 10/12 (83.3%) patients [10].

### Aspiration and biopsy of bone marrow

**Fig. 4:**
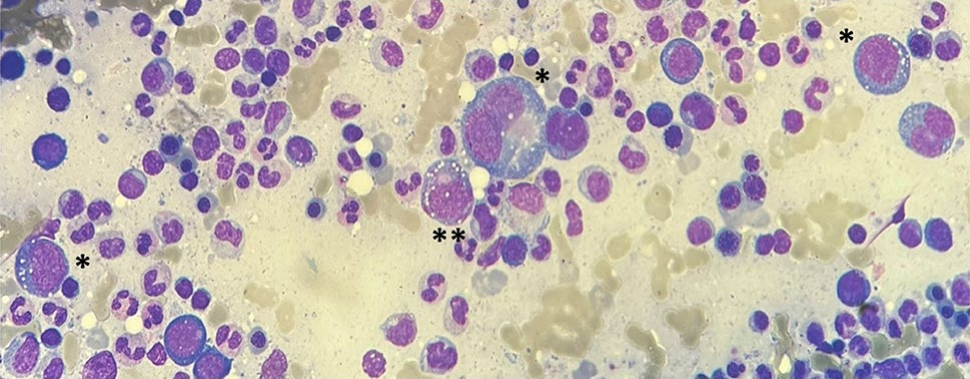
Bone aspirate smear showing vacuoles in erythroid precursors (signed with “*”) and in a myeloid precursor (signed with “**”)

**Fig. 5:**
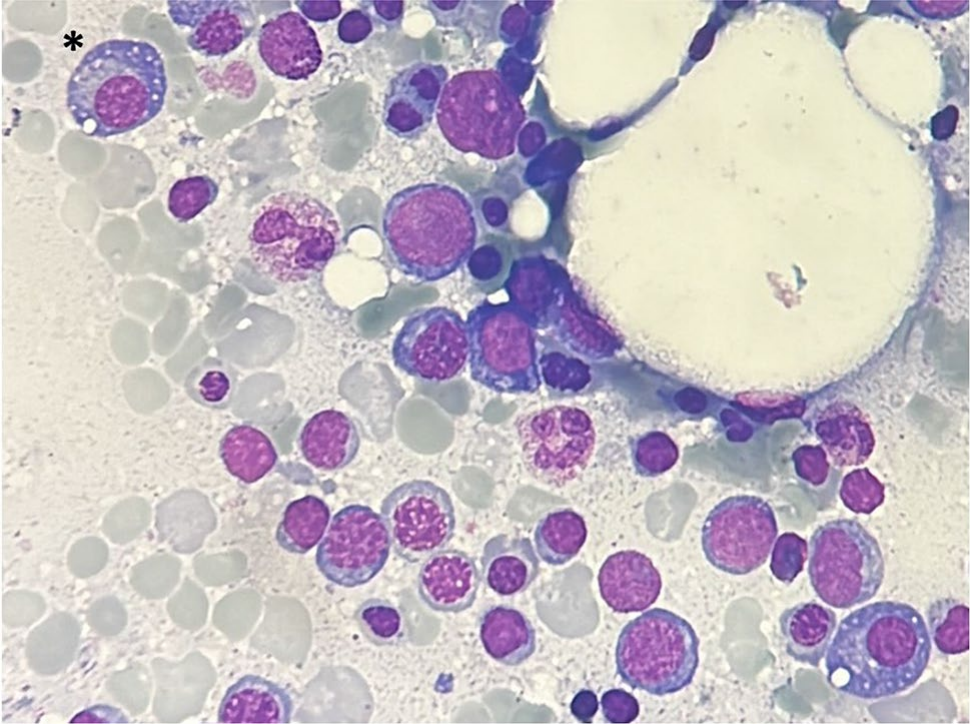
Bone aspirate smear from a VEXAS patient. Vacuoles are observed in plasma cells (signed with “*”)

The specific alterations resulting from the histopathologic examination of the bone marrow has a pivotal role during the diagnostic workup of VEXAS syndrome. In particular, bone aspirate smear can display pathognomonic alterations, especially cytoplasmic vacuolization of erythroid and myeloid precursors in almost all patients. Most of solid information regarding findings in bone marrow biopsy is drawn from Obiorah et al. [10]. Vacuoles are predominantly found in early precursors (blasts, promyelocytes, and pronormoblasts). Overall, 15% of myeloid and erythroid cells present vacuoles with an average of 5–7 vacuoles per cell. Vacuoles were also identified in eosinophils, monocytes, plasma cells, and megakaryocytes, although to a lesser degree. On the contrary, lymphocytes are generally devoid of vacuoles. Some degree of atypia or dyspoiesis were found in megakaryocytes and myeloid and erythroid precursors in nearly all bone marrow aspirates; however, a > 10% dysplasia in a lineage was seen only in patients with MDS [10]. Figures 4 and 5 represents examples of cells vacuolization in erythroid and myeloid precursors, but also in plasma cells in bone marrow smears from VEXAS patients.

Signs observed at bone marrow biopsy include characteristics reminding a MDS, such as hyperplasia, megakaryocytic atypias, and hemophagocytosis. According with Obiorah et al., bone marrow hypercellularity was found in 14/16 (87.5%) patients with a cellularity ranging from 25 to 100%. Myeloid hyperplasia was described in more than an half of the patients with myeloid:erythroid (M:E) ratios of 7:1 or greater, especially in patients complicated with MDS. Two out of the four patients with MDS had evidence of dyserythropoiesis highlighted by binucleation or multinucleation, nuclear budding, and/or marked megaloblastic changes; 3 of them evidenced dysmyelopoisis with hypogranular and/or hyposegmented precursors. The other patients showed milder myeloid hyperplasia or normal M:E ratios, but the cellularity might increase over time. Conversely, no patients demonstrated loss of cellularity without treatment. Megakaryocytes were increased in half of the patients and decreased in 2/16 patients. The percentage of bone marrow blasts was < 5% in all specimens [10].

Immunohistochemistry reveals myeloid cells with myeloperoxidase and CD68 positivity. Clonal B-cell populations indicative of monoclonal B-cell lymphocytosis were observed in 2/16 (13%) patients, with immunophenotypic features of chronic lymphocytic leukemia in both cases (CD51 and CD231). Megakaryocytic dysplasia with hypolobated or mononuclear megakaryocytes, megakaryocytes with separated nuclear lobes, and/or micromegakaryocytes characterised all the enrolled patients [10].

Obiorah et al. also observed that flow cytometry performed in 11 patients revealed absent B-cell precursors (< 1% of lymphocytes) in 10 (91%) cases, inverted CD4:CD8 T-cell ratio in 8 (73%) cases, and an aberrant CD56 expression on > 10% of monocytes in 6 out of 10 cases. An increased number of CD57 T cells (representing > 10% of lymphocytes) was identified in 10/11 cases; this percentage was even > 20% in 5/11 cases. The natural killer cells were decreased in 2 patients [10].

Disorders of plasma cells have been identified in up to 25%, including multiple myeloma and monoclonal IgG or IgA MGUS [10].

Regarding the need to repeat bone marrow aspirates, a progression in dyspoiesis seems to be anticipated by a worsening of peripheral cytopenia; conversely, a steady peripheral picture is associated to a stable bone marrow picture [10].

Of note, based on bone marrow evaluation Lacombe et al. [34] proposed easy-to-apply criteria capable of driving the decision toward genetic analysis of *UBA1* gene. In details, a threshold ≥ 10% of neutrophils precursors with at least one vacuole was highly associated to the identification of a *UBA1* mutation with a sensitivity of 100% and a specificity of 100%. Although intriguing, this accessible, cheap, and efficient way to select patients needing *UBA1* sequencing requires to be validated on a wider cohort of patients.

Electron microscopy can reveal myeloid cells undergoing death, vacuoles of lipid droplets and disordered cellular organelles, including degenerating mitochondria [1].

### Radiologic assessment

**Fig. 6:**
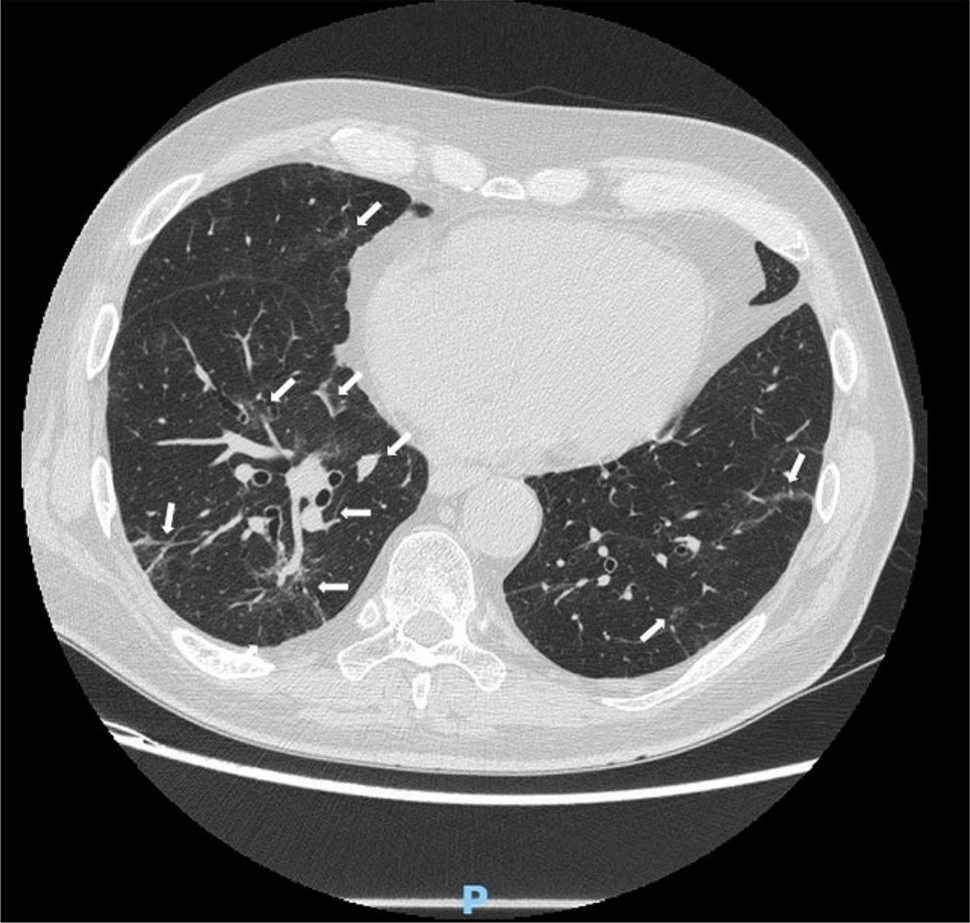
Multifocal lung inflammation in a patient with VEXAS syndrome. Peribronchial inflammation and multifocal ground glass (marked with arrows) in a patient with lung involvement is observed. The patient carries the p.Met41Val mutation on *UBA1* gene. Clinical picture is compatible with the cluster 2 according with Georgine-Lavialle et al. [5]

Chest X-ray eventually followed by computed tomography (CT) are essential to identify the lung inflammatory involvement and deep lymph node enlargement in VEXAS patients. Figure 6 shows an image from chest CT scan in a VEXAS patient with multifocal ground glass.

Angio-CT or magnetic resonance angiography may allow the identification of inflammatory involvement of the large vessels. Ultrasound with power Doppler may lead to the identification of thrombotic vascular events, eventually followed by angio-CT to rule out pulmonary thromboembolism; echocardiography may highlight cardiac involvement.

Positron emission tomography (PET) scan can highlight a diffuse enhancement of fludeoxyglucose 18F uptake in the bone marrow, spleen, and thyroid gland. Furthermore, PET scan can show mediastinal and hilar lymphadenopathy and nodules in lungs, pancreas, parotids, and other organs and may facilitate the identification of oncohematological VEXAS-associated disorders [22].

## Prognosis

The mortality rate is high in VEXAS syndrome. According to van der Made et al., it corresponds to 50% during an average follow-up of 3.96 years; Georgine-Lavialle et al. showed a 5-year survival rate of 63% [5, 22]. Death may be associated to either disease progression or adverse events related to treatment. Gastrointestinal involvement is particularly associated to mortality, especially in patients resembling polyarteritis nodosa and in patients with Behçet’s-like condition with MDS [5].

Georgin-Lavialle et al. divided patients with VEXAS syndrome in three clusters. Patients in cluster 1 (about 46.5% of all cases) show a mild-to-moderate disease with fewer constitutional symptoms such as recurrent fever episodes or weight loss, with less lung and lymph nodes involvement and less likely unprovoked thrombosis. In these cases, the median neutrophil counts and levels of C reactive protein are lower than in patients of other clusters. In this cluster the mutation p.Met41Leu (c.121A > C) is more frequent. Patients in cluster 2 show RP, gastrointestinal and heart involvement more frequently, along with the presence of pulmonary infiltrates. A lower platelet count, a high frequency of MGUS and MDS also characterise this second cluster. Cluster 3 is more frequently found among older patients and is characterised by a higher frequency of weight loss and cutaneous vasculitis, with less common RP and higher levels in values of C reactive protein. Analysis of the overall survival showed a higher mortality rate in cluster 2; the 5-year survival in this cluster was 62.7%, whilst cluster 1 and cluster 2 showed 87.4% and 93.1% 5-year survival, respectively. In particular, gastrointestinal involvement, the presence of lung infiltrates and mediastinal lymph node enlargement were significantly associated to mortality [5].

Notably, the overall survival rates seem to be not affected by any concomitant MSD. Conversely, a younger age at onset could account for a negative prognostic factor [22].

Multivariate analysis on 83 VEXAS patients with somatic *UBA1* mutations at p.Met41 showed an association of ear chondritis with increased survival, on the contrary, the development of transfusion dependence was independently associated to reduced survival. The same study evidenced that patients carrying the p.Met41Val mutations were more likely burdened by decreased survival [7]. On the other hand, non-Met41 variants have been suggested to induce a milder disease [35].

## Therapy evidence

There is a significant interindividual variability in treatments effectiveness in VEXAS syndrome. To date, treatments attempted include glucocorticoids, conventional disease-modifying antirheumatic drugs (cDMARDs), biotechnological agents targeting IL-1 and IL-6 and Janus kinase (JAK) inhibitors. Allogenic haematopoietic stem cell transplantation (HSCT) and future gene-editing therapies could be valid therapies to investigate.

Patients generally responds to high-dose corticosteroids, but flares are frequent after dosage tapering. Moreover, glucocorticoids side effects are frequent and further compromise an otherwise compromised clinical framework [5]. Therefore, although glucocorticoids are useful for initial disease control, transitioning to steroid-sparing therapies is necessary.

The use of the IL-1 receptor antagonist anakinra has led to a stabilization of the symptoms in some patients for 1–2 years but has also been reported to induce severe skin reactions in the site of injections [1]. However, the combination of anakinra with cyclosporin A has been proposed as a good option to avoid paradoxical skin flares with a systemic efficacy. However, some patients may experience significant neutropenia (800–900 cells/mm^3^) after the start of this treatment combination [36].

High serum IL-6 levels have been reported in patients with VEXAS syndrome [1]. In this regard, tocilizumab has been described to induce both modest effect and a good disease control [26, 37]. A study on three patients suggested that tocilizumab associated to low-dosage glucocorticoids may be an effective option in patients without severe hematologic abnormalities. In other cases, the combination of tocilizumab and methotrexate has further improved the therapeutic role of IL-6 inhibition [38]. Nevertheless, unresponsive patients have also been reported [26, 39] and adverse events may be frequent in VEXAS syndrome. In particular, tocilizumab treatment has been found associated to neutropenia, viral infections reactivations (especially herpes zoster virus), and severe gastrointestinal side effects in VEXAS patients [38, 40]. Regarding patients with gastrointestinal involvement, tocilizumab treatment seems to be a risk factor for perforation of the jejunum or ileum in the site of diverticulitis. Consequently, despite the tocilizumab effectiveness reported in VEXAS syndrome [37, 40], a particular attention should be given to this subgroup of patients.

Among anti-IL-6 agents, it is reported a good response in a case treated with siltuximab [17].

The Janus kinases (JAK) inhibitors have also been used in VEXAS syndrome, leading to discrepant results ranging from lack of response to complete control of inflammatory manifestations. The JAK1 and JAK2 inhibitor ruxolitinib has been reported to be more effective than other JAK inhibitors, with a clinical and laboratory response obtained in a half of patients within one month and in more than 80% of patients at the 3-month assessment. On the other side, clinical and biological response were observed in a remarkably lower percentage among patients treated with tofacitinib, baricitinib and upadacitinib [41]. Regarding the haematological picture, JAK inhibitors do not seem to control peripheral cytopenia [26]. Future studies should be conducted to better highlight the real role of JAK inhibitors in VEXAS patients.

The use of the hypomethylating agent azacytidine in VEXAS syndrome has been also suggested as an effective treatment strategy, especially in patients with concomitant MDS, those without aplastic bone marrow and patients with a concomitant *DNMT3A* mutation. In this regard, DNMT3A could sensitize hematopoietic cells to azacytidine [9, 42].

Based on a recent case report, abatacept associated with 5 mg/day prednisolone has also been described as useful in controlling clinical manifestations of VEXAS syndrome during a 30-month follow-up period. The patient had shown to be unresponsive to the conventional immunosuppressants methotrexate, azathioprine, and cyclosporine as well as to the anti-TNF agents adalimumab, etanercept and golimumab [43].

HSCT may represent a further treatment opportunity especially for severe or multiresistant patients. Indeed, according with van der Made et al. [22], 50% of the patients becomes therapy-refractory over time. In these cases, an early diagnosis is important to minimize physical deterioration prior to the transplantation. Diarra et al. presented 6 patients with VEXAS syndrome who underwent allogenic stem cells transplantation after a multiple treatment refractoriness. Five of them were still in complete remission at the last assessment, whilst one patient died due to infectious complications [39]. Other additional two cases undergoing up-front allo-HSCT have been described, with excellent results at 4-month and 9-month assessment, respectively [44, 45].

## Conclusions

VEXAS syndrome is a very interesting clinical condition in several respects. First, it accounts for the paradigm of monogenic autoinflammatory diseases with delayed onset during adulthood. Moreover, it represents an example of how a genetic mosaicism results in the development of a systemic inflammatory condition capable of involving all tissues and organs. Furthermore, VEXAS syndrome is probably not an exceptionally rare condition and may represent only the first example of systemic autoinflammatory diseases drawing its origin in bone marrow disorders. For all these reasons, VEXAS syndrome will be the subject of intensive research in the coming years to comprehensively evaluate the genetic variants capable of causing the syndrome, including any association with coexisting mutations in other genes, and to better understand clinical behaviour, outcome, and the most appropriate therapeutic approach. In this regard, it is useful to join forces internationally and collect sufficient data and obtain solid and definitive results. To this end, the international registry dedicated to VEXAS syndrome, supported by the AutoInflammatory Disease Alliance (AIDA) Network, has been developed and is already enrolling patients [46].

VEXAS syndrome should be strongly considered in each patient with unexplained systemic inflammatory condition, especially when associated with peripheral cytopenia and/or increased mean corpuscular volume and/or other hematologic disorders. The syndrome should be considered as a possible diagnosis whenever recurrent fevers, neutrophilic dermatosis, RP, and other autoinflammatory symptoms occur in adult patients. In this regard, multidisciplinary collaboration is essential for the diagnosis and management of a potentially very challenging clinical entity.
